# Monitoring microbial responses to ocean deoxygenation in a model oxygen minimum zone

**DOI:** 10.1038/sdata.2017.158

**Published:** 2017-10-31

**Authors:** Steven J. Hallam, Mónica Torres-Beltrán, Alyse K. Hawley

**Affiliations:** 1 Department of Microbiology and Immunology, University of British Columbia, Vancouver, British Columbia, Canada V6T 1Z3; 2 Peter Wall Institute for Advanced Studies, University of British Columbia, Vancouver, British Columbia, Canada V6T 1Z2; 3 Genome Science and Technology Program, University of British Columbia, Vancouver, British Columbia, Canada V6T 1Z4; 4 Graduate Program in Bioinformatics, University of British Columbia, Vancouver, BC, Canada V6T 1Z4; 5 ECOSCOPE Training Program, University of British Columbia, Vancouver, British Columbia, Canada V6T 1Z4

**Keywords:** Marine chemistry, Microbial ecology

## Abstract

Today in *Scientific Data,* two compendia of geochemical and multi-omic sequence information (DNA, RNA, protein) generated over almost a decade of time series monitoring in a seasonally anoxic coastal marine setting are presented to the scientific community. These data descriptors introduce a model ecosystem for the study of microbial responses to ocean deoxygenation, a phenotype that is currently expanding due to climate change. Public access to this time series information is intended to promote scientific collaborations and the generation of new hypotheses relevant to microbial ecology, biogeochemistry and global change issues.

*“I have long felt that the future of molecular biology lies in the extension of research to other fields of biology, notably development and the nervous system. This is not an original thought, because as you well know, many other molecular biologists are thinking in the same way. The great difficulty about these fields is that the nature of the problem has not yet been clearly defined, and hence the right experimental approach is not known.”*_*Sydney Brenner circa 1963*_

The ocean is changing. In addition to becoming warmer and more acidic, dissolved O_2_ concentrations within coastal and interior regions are decreasing, resulting in oxygen minimum zone (OMZ) expansion^1–3^. Ocean deoxygenation can negatively impact ecosystem functions and services through changes in food web structure and biological diversity^4,5^. For example, over the last 50 years, Northeastern subarctic Pacific (NESAP) surface waters have warmed and freshened due to climate change making the upper ocean more buoyant with concomitant stratification^6^. This process is associated with a slowing down of the North Pacific Intermediate Water (NPIW) formation and persistent deoxygenation within the ocean’s interior. Oxygen concentrations within the core of the NESAP OMZ have decreased by 22% and the hypoxic boundary (defined as 60 μmol/kg) has shoaled upwards from 400 to 300 m. In coastal waters west of Vancouver Island (BC, Canada), wind patterns and the divergence of the surface waters to the north and south create an upwelling regime that brings up subsurface waters from depths of 100 to 250 m^6^. Continued OMZ expansion in the NESAP has the potential to transport oxygen-depleted waters into coastal regions adversely effecting fisheries productivity. Indeed, fish and crab kills induced by water column deoxygenation have already been observed along the coast of Oregon, Washington, and British Columbia^7^. These ecological impacts represent a recurring theme throughout the global ocean that transcends the anthropogenic boundaries of single nations or states.

As water column oxygen levels decline, less energy is available to higher trophic levels, increasing the role of microbial metabolism in nutrient and energy cycling through the use of alternative terminal electron acceptors (TEAs)^4^. This results in fixed nitrogen loss and the production of greenhouse gases like nitrous oxide (N_2_O) and methane (CH_4_) that influence global warming^8,9^. Both of these gases contribute to warming by increasing the amount of solar energy that is absorbed by the planet measured as radiative forcing in Watts per square meter. However, the global warming potential (GWP) of N_2_O and CH_4_ varies substantially from the most common greenhouse gas, carbon dioxide (CO_2_) by 300- and 30-fold respectively (based on one-hundred year atmospheric residence times). Current research efforts are defining the interaction networks underlying microbial metabolism in OMZs and generating new insights into coupled biogeochemical processes in the ocean driving climate active trace gas cycling^10–14^. However, many open questions remain regarding the future oxygenation status of the ocean and the response of microorganisms at the individual, population and community levels to OMZ expansion. This has important implications for current models of climate forcing and nutrient cycling in coastal and open ocean waters.

Model organismal systems such as *Escherichia coli, Saccharomyces cerevisiae, Caenorhabditis elegans* and *Drosophila melanogaster* have provided unifying frameworks for community-driven research that have revealed fundamental organizing principles of life and the dynamics of cellular networks. The development of similar frameworks in which to evaluate ecological interactions and response to perturbation at ecosystem scales is becoming increasingly tractable with the advent of high-throughput sequencing and mass spectrometry platforms that reveal the hidden metabolic powers of uncultivated microbial communities. In 2014, the Scientific Committee on Oceanic Research (SCOR) initiated Working Group 144 to define model ecosystems and standards for combined process rate and molecular data collection needed for effective cross-scale comparisons and enhanced forecasts of ocean deoxygenation (http://www.scor-int.org/SCOR_WGs_WG144.htm).

Today in *Scientific Data,* two compendia of geochemical and multi-omic sequence information from Saanich Inlet are provided in support of SCOR 144 activities and aspirations^15,16^. Saanich Inlet is a seasonally anoxic fjord on the east coast of Vancouver Island British Columbia, Canada. A recurring seasonal development of water column anoxia followed by deep water renewal enables spatiotemporal profiling across a wide range of water column redox states including conditions associated with anoxic and sulfidic OMZs^17^ making Saanich Inlet a model ecosystem for studying microbial responses to ocean deoxygenation including foundational studies on seasonal stratification and deep water renewal^18,19^, trace metal cycling, methane oxidation^20–22^, nitrification^23,24^, and chemoautotrophic carbon fixation^25^. The close proximity of the Department of Fisheries and Oceans (DFO) Institute for Ocean Sciences (IOS, http://www.pac.dfo-mpo.gc.ca/sci/sci/facilities/ios_e.htm) and the Victoria Experimental Network Under the Sea (VENUS) cabled observatory (http://www.venus.uvic.ca/) infrastructures facilitate sample and data collection. Moreover, taxonomic and genomic surveys of the Saanich Inlet water column have identified conserved patterns of microbial community composition extensible to other coastal, open ocean and enclosed basin OMZs^26–28^.

The combined use of geochemical and multi-omic sequence information have led to new insights into coupled biogeochemical cycling of C, N and S between key microbial players and the development of a predictive ecosystem model describing the flow of multi-omic sequence information and process rates along eco-thermodynamic gradients^11,12,14^. Thus, time-series data from Saanich Inlet provides a community-driven framework for observing and predicting microbial community repsonses to ocean deoygenation across multiple scales of biological organization. Readers are encouraged to use this information as a resource for comparative studies and a source of inspiration for developing reproducible hypothesis-driven research in Saanich Inlet and beyond.

## Additional Information

**How to cite this article:** Hallam, S. J. *et al.* Monitoring microbial responses to ocean deoxygenation in a model oxygen minimum zone. *Sci. Data* 4:170158 doi: 10.1038/sdata.2017.158 (2017).

**Publisher’s note:** Springer Nature remains neutral with regard to jurisdictional claims in published maps and institutional affiliations.
